# RETRACTION: A Meta‐Analysis of the Risk Factors for Surgical Site Infection in Patients With Colorectal Cancer

**DOI:** 10.1111/iwj.70266

**Published:** 2025-02-25

## Abstract

**RETRACTION:** ChenY. , GuoH. , GaoT. , YuJ. , WangY. , and YuH. , “A Meta‐Analysis of the Risk Factors for Surgical Site Infection in Patients With Colorectal Cancer,” International Wound Journal 21, no. 2 (2024): e14459, 10.1111/iwj.14459.PMC1082852937904719

The above article, published online on 31 October 2023, in Wiley Online Library (http://onlinelibrary.wiley.com/), has been retracted by agreement between the journal Editor in Chief, Professor Keith Harding; and John Wiley & Sons Ltd. Following an investigation by the publisher, all parties have concluded that this article was accepted solely on the basis of a compromised peer review process. In addition, the investigation found significant textual overlap between the methods and discussions sections of this article and another article by different authors [1]. The editors have therefore decided to retract the article. The authors did not respond to our notice regarding the retraction.

**Reference:**

[1] XuZ. , QuH. , KananiG. , GuoZ. , RenY. , and ChenX. , “Update on Risk Factors of Surgical Site Infection in Colorectal Cancer: a Systematic Review and Meta‐Analysis,” International Journal of Colorectal Disease 35 (2020): 2147–2156, 10.1007/s00384-020-03706-8.32748113

**RETRACTION:** Y. Chen , H. Guo , T. Gao , J. Yu , Y. Wang , and H. Yu , “A Meta‐Analysis of the Risk Factors for Surgical Site Infection in Patients With Colorectal Cancer,” International Wound Journal 21, no. 2 (2024): e14459, 10.1111/iwj.14459.PMC1082852937904719

The above article, published online on 31 October 2023, in Wiley Online Library (http://onlinelibrary.wiley.com/), has been retracted by agreement between the journal Editor in Chief, Professor Keith Harding; and John Wiley & Sons Ltd. Following an investigation by the publisher, all parties have concluded that this article was accepted solely on the basis of a compromised peer review process. In addition, the investigation found significant textual overlap between the methods and discussions sections of this article and another article by different authors [1]. The editors have therefore decided to retract the article. The authors did not respond to our notice regarding the retraction.

## Reference

[1] Z. Xu , H. Qu , G. Kanani , Z. Guo , Y. Ren , and X. Chen , “Update on Risk Factors of Surgical Site Infection in Colorectal Cancer: a Systematic Review and Meta‐Analysis,” International Journal of Colorectal Disease 35 (2020): 2147–2156, 10.1007/s00384-020-03706-8.32748113

